# O-GlcNAcylation, contractile protein modifications and calcium affinity in skeletal muscle

**DOI:** 10.3389/fphys.2014.00421

**Published:** 2014-10-30

**Authors:** Caroline Cieniewski-Bernard, Matthias Lambert, Erwan Dupont, Valérie Montel, Laurence Stevens, Bruno Bastide

**Affiliations:** ^1^Université LilleLille, France; ^2^EA4488, APMS, URePsss, Université de Lille 1Villeneuve d'Ascq, France

**Keywords:** O-GlcNAcylation, phosphorylation, O-GlcNAcylation/phosphorylation interplay, contractile proteins, MLC2, contractile properties, sarcomeric structure

## Abstract

O-GlcNAcylation, a generally undermined atypical protein glycosylation process, is involved in a dynamic and highly regulated interplay with phosphorylation. Akin to phosphorylation, O-GlcNAcylation is also involved in the physiopathology of several acquired diseases, such as muscle insulin resistance or muscle atrophy. Recent data underline that the interplay between phosphorylation and O-GlcNAcylation acts as a modulator of skeletal muscle contractile activity. In particular, the O-GlcNAcylation level of the phosphoprotein myosin light chain 2 seems to be crucial in the modulation of the calcium activation properties, and should be responsible for changes in calcium properties observed in functional atrophy. Moreover, since several key structural proteins are O-GlcNAc-modified, and because of the localization of the enzymes involved in the O-GlcNAcylation/de-O-GlcNAcylation process to the nodal Z disk, a role of O-GlcNAcylation in the modulation of the sarcomeric structure should be considered.

## O-GlcNAcylation, an atypical glycosylation

**Figure 1:**
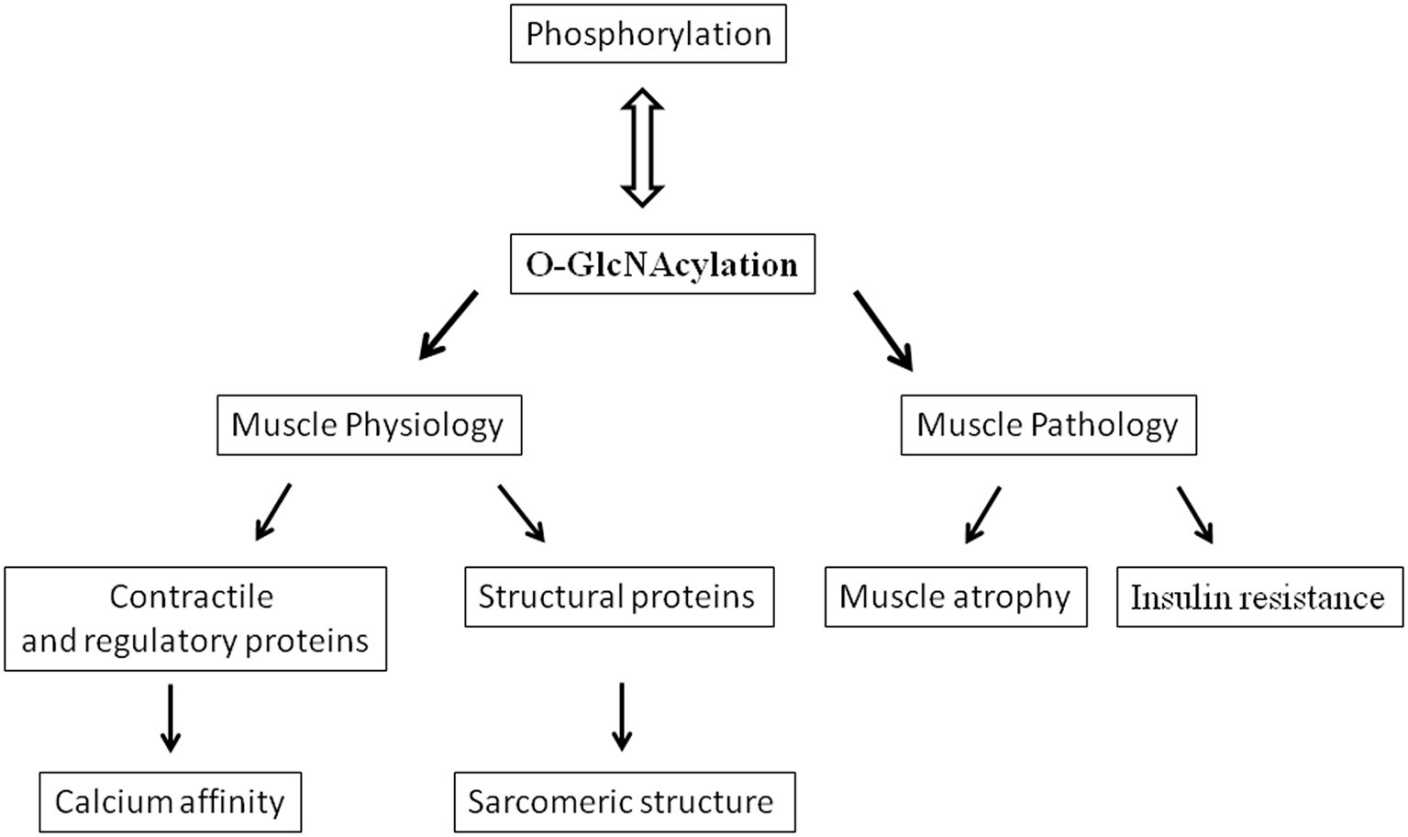
**Diagram showing various effects and implications of O-GlcNAcylation in skeletal muscle**.

Nowadays, it is well admitted that the phosphorylation does not act alone in the fine modulation of numerous cellular processes, but rather, presents a dynamic and highly regulated interplay with an atypical glycosylation, the O-linked N-acetyl-glucosaminylation (termed O-GlcNAcylation), occurring on nuclear, cytoplasmic and mitochondrial proteins (Hart et al., 2007; Cao et al., 2013; Johnsen et al., 2013). This minireview is based on significant references focused on the recent advancements concerning the link between O-GlcNAcylation, contractile proteins and calcium affinity in skeletal muscle (Figure 1).

The O-GlcNAcylation of proteins results from the transfer of N-acetyl-β-D-glucosamine from the high energy donor substrate UDP-GlcNAc (synthesized through the hexosamine biosynthesis pathway) onto the hydroxyl group of serine and threonine residues of target proteins by the uridine diphospho-N-acetyl glucosaminyl transferase (O-GlcNAc transferase or OGT). The β-N-acetylglucosaminidase (O-GlcNAcase or OGA) catalyzes the removal of O-GlcNAc residues from proteins (Dong and Hart, 1994; Gao et al., 2001; Wells et al., 2002). Thus, like phosphorylation, the addition/removal of GlcNAc moieties on the proteins results from the concerted action of two antagonist enzymes. Reversible, O-GlcNAcylation is highly dynamic, and responds rapidly to changes in environmental conditions (Hart et al., 2007). Since OGT is coded from only one gene (Kreppel et al., 1997), the regulation of its activity, its localization in cell, or its specificity toward protein targets are ensured by targeting proteins, such as protein phosphatase 1 (PP1), Milton (OIP106), p38MAP kinase, myosin phosphatase 1 (MYPT1) or peroxisome-proliferator-activated receptor-co-activator-1alpha (PGC1α), transiently associated with OGT through tetratricopeptide repeats (TPR domains) at the N-termini of the transferase (Iyer and Hart, 2003; Iyer et al., 2003; Wells et al., 2004; Cheung and Hart, 2008; Cheung et al., 2008; Housley et al., 2009). In the same way, the OGA could also be associated with other proteins like calcineurin or heat shock proteins among others (Wells et al., 2001). Moreover, an unusual association of the two opposing OGA/OGT was described, forming a single O-GlcNAczyme complex (Whisenhunt et al., 2006).

The analysis of O-GlcNAc pattern, the quantification of variation of O-GlcNAcylation on proteins and the identification of the glycosylated sites are crucial for the understanding of the role of this atypical glycosylation. Methodological approaches include western blot analyses using antibodies directed against O-GlcNAc moieties or lectins (Zachara et al., 2011), or the labeling of O-GlcNAcylated proteins with galactosyltransferase and coupling of different kind of substrates (Zachara et al., 2011). Moreover, the identification and mapping of O-GlcNAc modification sites have been at the origin of several technical developments during the 10 last years (Ma and Hart, 2014 for review).

O-GlcNAcylation has been shown to be involved in almost all cellular processes, including signal transduction, protein degradation or regulation of gene expression (Hart et al., 2011; Bond and Hanover, 2013). O-GlcNAcylation has been also demonstrated to act as an inducible, cytoprotective stress response. Indeed, increase in O-GlcNAcylation of nucleocytoplasmic proteins protects cells with an induction of heat shock proteins (Zachara et al., 2004). In cardiomyocytes, O-GlcNAcylation can attenuate oxidative stress through inhibition of calcium overload and ROS generation (Ngoh et al., 2010). Studies performed with OGT and OGA knockout mice have demonstrated that O-GlcNAc is crucial for life since O-GlcNAcylation was essential for embryonic stem cell viability and is implicated in the aging process, elevation of O-GlcNAcylation being altered in different tissues of different ages (Shafi et al., 2000; Yang et al., 2012). Moreover, many reports demonstrate that O-GlcNAcylation might play a role in the physiopathology of several acquired diseases, such as cancer, cardiovascular diseases, neurodegenerative diseases, Alzheimer or type II diabetes (Lefebvre et al., 2010; Slawson et al., 2010; Nakamura et al., 2012; Bond and Hanover, 2013; Ma and Hart, 2013; Ma and Vosseller, 2013; Förster et al., 2014). Indeed, increased O-GlcNAcylation is closely linked to insulin resistance and hyperglycemia-induced glucose toxicity.

## O-GlcNAcylation, skeletal muscle, and insulin-resistance

The concept of a role of O-GlcNAcylation in skeletal muscle has emerged from studies considering muscle as one of the crucial insulin-sensitive tissue; indeed, skeletal muscle is responsible for more than 80% of insulin-stimulated glucose uptake in humans. In the past decades, studies in rodents (Hawkins et al., 1997a,b) suggested a correlation between the development of insulin resistance and increased UDP-GlcNAc concentrations in muscle. Thus, raising O-GlcNAc level in skeletal muscle has been demonstrated to induce insulin resistance (Arias and Cartee, 2005, Figure 1), while coinfusion of insulin and glucosamine, increasing UDP-GlcNAc, enhances the O-GlcNAc modification on numerous unidentified skeletal muscle proteins (Yki-Jarvinen et al., 1998). Moreover, transgenic mice overexpressing GLUT1 in skeletal muscle was insulin resistant and exhibit chronically increased glucose flow and increased UDP-GlcNAc concentrations in muscle (Buse et al., 1996); indeed, 2–5% of the glucose entering into the cell is directed to the hexosamine biosynthesis pathway, leading to the synthesis of UDP-GlcNAc, the donor for O-GlcNAcylation. More recently, it has been suggested that mitochondrial and contractile dysfunctions, observed in the development of Type 2 diabetes, were linked to the increase in O-GlcNAcylation level (Johnsen et al., 2013). Thus, in rats artificially selected for Low Running Capacity (LCR rats), predisposed to becoming obese, and developing insulin resistance and cardiovascular dysfunction, the increase in O-GlcNAcylation on mitochondrial proteins and SERCA was associated with mitochondrial dysfunction and changes in contractile properties (Koch et al., 2012). In the LCR heart myocytes, the cardiovascular dysfunction could be attributed to the decline in the contractility, correlated to the impaired intracellular Ca^2+^ handling and signaling (Koch et al., 2012). Hu et al. (2005) demonstrated also the correlation between O-GlcNAcylation and diabetes since they demonstrated that adenoviral transfer of OGA into the myocardium of streptozotocin induced diabetic mice, reversed the excessive O-GlcNAc modifications associated with diabetes, particularly in the contractile dysfunctions and Ca^2+^ handling capacities (Hu et al., 2005). The decline in contractibility could be related to the decrease in calcium affinity and sensitivity reported in skinned cardiac fibers in presence of GlcNAc (Ramirez-Correa et al., 2008) but rather involved changes in expression of SERCA (Hu et al., 2005; Johnsen et al., 2013). This role of O-GlcNAcylation should be considered also in skeletal muscle since dysfunction of contractibility as well as the Ca^2+^ handling were also measured in skeletal muscle of rat models of diabetes (Eshima et al., 2013), via impaired SERCA and Glut 4 (Safwat et al., 2013).

## O-GlcNAcylation/phosphorylation interplay and skeletal muscle contractile activity

Phosphorylation was well-admitted for a long time to regulate many key processes in muscle physiology. Among them, phosphorylation is a key regulator of the intracellular signaling pathways involved in muscle mass and phenotype adaptation to physiological demands. Moreover, phosphorylation is involved in myofibrillar physiology, such as muscular contraction or cellular structuration through the modulation of protein-protein interactions. Indeed, phosphorylation has been demonstrated to play a crucial role in the regulation of the contractile properties in striated muscle, but recent reports suggested that O-GlcNAcylation might play a role as important as phosphorylation in muscle physiology. Thus, many proteins of muscle proteome have been identified to be O-GlcNAc modified (Cieniewski-Bernard et al., 2004, 2012; Hedou et al., 2007; Ramirez-Correa et al., 2008). Among them, several key contractile proteins of striated muscle are concerned, i.e., myosin heavy chains (slow MHCI as well as the fast isoforms MHCIIA and MHCIIB), myosin light chains (essential MLC or MLC1 and regulatory MLC or MLC2), actin, both α and β isoforms of tropomyosin as well as isoforms of TnI and TnT. It is noteworthy that it is not known whether TnC, the calcium-sensor, bears an O-GlcNAc moiety or not.

While contraction is triggered by calcium release, it is regulated by several myofilament proteins such as the regulatory myosin light chain (termed MLC2). MLC2 phosphorylation is not essential for skeletal muscle contraction but is an important regulatory mechanism since it can produce changes in thick filament structure and enhance crossbridge attachment (Szczesna et al., 2002). For instance, phosphorylation of MLC2, catalyzed by a Ca^2+^/calmodulin-dependant MLC kinase, increases the force development at submaximal calcium concentration, conferring a higher calcium sensitivity to the fibers (Persechini et al., 1985; Stephenson and Stephenson, 1993; Sweeney et al., 1993; Szczesna et al., 2002).

Our recent data highlight the key role of O-GlcNAcylation as a modulator of skeletal muscle contractile activity, in particular on the calcium activation properties (Hedou et al., 2007; Cieniewski-Bernard et al., 2012). Indeed, muscle skinned fibers, when exposed to N-acetyl-D-glucosamine, present a reversible decrease in calcium sensitivity and affinity, whereas the cooperativity within the thin filament was not changed (Hedou et al., 2007). This modulation probably involved disruption of protein-protein interactions through O-GlcNAc moieties. Interestingly, a similar effect was observed in rats as well as in human skinned fibers (Cieniewski-Bernard et al., 2009) and also measured in cardiac trabeculae (Ramirez-Correa et al., 2008).

Further experiments performed after the pharmacological increase of O-GlcNAcylation level of contractile protein glycosylation, using PUGNAc or Thiamet G, two inhibitors of OGA (Gloster and Vocadlo, 2010), leads to an increase of calcium affinity on slow soleus skinned fibers (Cieniewski-Bernard et al., 2012). Several regulatory contractile proteins, predominantly fast isoforms, presented a drastic increase in their O-GlcNAc level. Since the only slow isoform of contractile protein (and so the more representative isoforms in the slow skeletal muscle) presenting an increase of O-GlcNAc level was the myosin regulatory light chains MLC2, the effect of enhanced O-GlcNAcylation pattern on calcium activation parameters of slow soleus fibers was attributed to the increase of the O-GlcNAcylation of sMLC2 (Cieniewski-Bernard et al., 2012).

All these data closely linked O-GlcNAcylation to the modulation of contractile activity of skeletal muscle, a decrease in O-GlcNAcylation being associated to a decrease in calcium affinity and reciprocally (Figure 1). Moreover, analysis of the proteins presenting changes in their O-GlcNAc level states suggests that MLC2 could play an important role in this modulation. Since MLC2 was identified to be O-GlcNAcylated (Hedou et al., 2007), and because of the potential antagonism between phosphorylation and O-GlcNAcylation, i.e., the potential O-GlcNAcylated site corresponds to the only site of phosphorylation in MLC2 located in Ser 14 for the rat slow MLC2 isoform (Blumenthal and Stull, 1980; Ramirez-Correa et al., 2008), the O-GlcNAcylation should be considered as a potential new mechanism that could modulate the contractile properties of skeletal muscle as well as the phosphorylated states of MLC2.

## O-GlcNAcylation/phosphorylation interplay, and skeletal muscle contractile dysfunction in atrophied muscle

Muscle atrophy characterized skeletal muscle adaptation to a large variety of disuse conditions (immobilization, microgravity, bed rest, or nerve injury). This atrophy results from a reduction in fiber diameter, protein content and is associated with slow to fast phenotype transitions accompanied by functional changes such as loss in force and increased fatigability (Baldwin et al., 1996; Fitts et al., 2000; Fluck and Hoppeler, 2003; Mounier et al., 2009). Moreover a role of O-GlcNAcylation in the development of muscle atrophy has been suggested (Cieniewski-Bernard et al., 2006; Figure 1). Indeed, a correlation between variations in O-GlcNAcylation levels and the development of atrophy after hindlimb unloading was shown, suggesting that O-GlcNAc variations could control the muscle protein homeostasis (Cieniewski-Bernard et al., 2006). In particular, it was suggested that O-GlcNAcylation should be implicated in the regulation of muscular atrophy as a protective mechanism against proteasomal degradation (Cieniewski-Bernard et al., 2006). It was also recently described that muscle-specific overexpression of NCOATGK, a splice variant of O-GlcNAcase, induces skeletal muscle atrophy (Huang et al., 2011).

We have demonstrated that the slow-to-fast transition (at MHC and MLC2 level) concomittantly to soleus muscle atrophy was correlated with an increase of the global level of MLC2 phosphorylation in rat hindlimb unloading model (Bozzo et al., 2005) as well as in human patients who were subjected to 2-months Bed Rest (Stevens et al., 2013). However, similar variations in MLC2 phosphorylation have been observed when clenbuterol was administrated to rats; in such case, muscle hypertrophy was associated to slow-to-fast transition in rats (Bozzo et al., 2003).

Thus, the slow-to-fast MLC2 transition was associated to an increase of phosphorylation level of the two isoforms regardless of whether hypertrophy or atrophy develops. Moreover, although numerous data argue that MLC2 phosphorylation lead to higher calcium sensitivity in skeletal muscle, an increase in MLC2 phosphorylation was measured after muscle atrophy whereas the calcium sensitivity decreases (Bozzo et al., 2003). Similar data were obtained from phosphoproteome analysis in human aged muscle (Gannon et al., 2008). These data could indicate that the decrease in calcium sensitivity occurring during hindlimb unloading could involve another type of regulation than classical phosphorylation. Since we demonstrated that a decrease in calcium affinity was measured when O-GlcNAcylation is reduced, and since a decrease in O-GlcNAcylation is observed in atrophied muscle, one hypothesis is that O-GlcNAcylation might be involved in this alteration.

To support this hypothesis, the variation of O-GlcNAcylation level on MLC2, as well as its interplay with phosphorylation, was investigated in atrophied soleus muscle (Cieniewski-Bernard et al., in press). Interestingly, an antagonism between phosphorylation and O-GlcNAcylation was demonstrated on the slow MLC2 in a rat model of hindlimb unloading, largely used to induce functional atrophy of antigravitary muscles such as soleus (Cieniewski-Bernard et al., in press), since a decrease in MLC2 O-GlcNAcylation was measured in atrophied soleus associated to the increase in phosphorylation states as previously described. More importantly, the use of Phos-Tag acrylamide gels allowed the authors to analyze the O-GlcNAcylation state on each phosphorylated form of MLC2 (Cieniewski-Bernard et al., in press). It was demonstrated that the two post-translational modifications were mutually exclusive, and that this interplay was closely associated to load because of the reversibility of these processes occurring during reloading. Interestingly, it was demonstrated that the enzymes involved in the phosphorylation/dephosphorylation and O-GlcNAcylation/de-O-GlcNAcylation processes were associated within a multi-enzymatic complex, and were localized to the nodal Z disk region of the sarcomere. All these results are in favor of an interplay between phosphorylation and O-GlcNAcylation on MLC2, probably through the same site.

This interplay between phosphorylation and O-GlcNAcylation in the fine modulation of MLC2 activity was also observed in humans (Stevens et al., 2013). Indeed, the post-translational modifications of MLC2 were investigated in soleus biopsies obtained from 60-days Bed-Rest female subjects. In this cohort, several groups were formed: 60-days Bed Rest (BR), BR + Exercice (combined aerobic and resistive exercises), and BR + Nutritional protocol (leucine and valine diet). The slow-fast phenotype transition of MLC2 was associated to an increase of the phosphorylation states of slow and fast isoforms MLC2 while the global MLC2 glycosylation level was decreased. Interestingly, aerobic and resistive exercises, which preserved muscles from BR changes (and so slow-to-fast transition), also prevented this antagonism.

Taken together, all these data clearly corroborated the interplay between phosphorylation and O-GlcNAcylation on MLC2, in animal and in human models of functional atrophy. Indeed, the decrease in calcium sensitivity as well as calcium affinity measured in skinned fibers of atrophied muscle soleus cannot be obviously explained by the increase in MLC2 phosphorylation state, as it was previously described (Bozzo et al., 2003). Since previous experiments have demonstrated that decrease in O-GlcNAcylation was associated to a decrease in calcium sensitivity and affinity, whereas its phosphorylated state increased in atrophied muscle, MLC2 O-GlcNAcylation state could be predominantly involved in the alteration of contractile parameters observed in atrophied soleus. In contrast, the MLC2 phosphorylation might be rather associated to phenotype transition than to changes in contractile parameters in atrophied muscle. Interestingly, some data support for a structural role of MLC2 phosphorylation: MLC2 phosphorylation induces stress fiber assembly in nonmuscle cells (Katoh et al., 2001) and mediates sarcomere organization during hypertrophic growth in cardiac muscle (Aoki et al., 2000).

However, further functional experiments need to be performed to demonstrate unambiguously that the phosphorylation/O-GlcNAcylation balance of MLC2 might be directly involved in the decrease in calcium sensitivity and affinity observed in atrophied muscles. While recent reports focused on MLC2, other regulatory contractile proteins have been demonstrated to be O-GlcNAcylated such as Tropomyosin, TnT and TnI (Cieniewski-Bernard et al., 2012). We cannot totally exclude that the glycosylation state of these proteins nor that unidentified O-GlcNAc proteins (more particularly, TnC is one of them) might play a role in the modulation of contractile activity.

## Myofibrillar proteins O-GlcNAcylation and perspectives

From the data described above, it seems very likely that O-GlcNAcylation plays a major role in modulating the contractile activity in skeletal muscle. However, the role of the O-GlcNAcylation might also concern other mechanisms involved in the sarcomeric structure. The preferential localization of OGT and OGA in the sarcomere, more particularly at the Z disk region (Cieniewski-Bernard et al., in press), argue for an important role of O-GlcNAcylation in this nodal region that could influence mechanisms other than the modulation of the contractile activity. Indeed, it was demonstrated that myosin, actin but also key proteins involved in the sarcomeric structure (desmin, actinin, αB-crystallin, and ZASP) were modified by O-GlcNAc moieties (Cieniewski-Bernard et al., 2012; Leung et al., 2013). Interestingly, we demonstrated that the O-GlcNAc sites on myosin were localized in a region involved in polymerization and interaction of myosin with proteins partner such as myomesin, M-protein or titin (Hedou et al., 2009) supporting a role for O-GlcNAcylation in the organization of the sarcomere (Figure 1). This hypothesis is underlined by the fact that numerous structural proteins are modified by O-GlcNAc: beta3 integrin (Ahmad et al., 2006), vinculin (Laczy et al., 2010), spectrin (Zhang and Bennett, 1996), alphaB-crystallin (Roquemore et al., 1996), laminin (Kwak et al., 2010), or cytokeratin (Ku and Omary, 1995), and variation of O-GlcNAcylation of the intermediate filaments has been demonstrated to lead to defective phosphorylated states and so to polymerization (Farah and Galileo, 2008; Slawson et al., 2008). Moreover, O-GlcNAcylation modulates organization and solubilization of cytokeratin (Rotty et al., 2010; Srikanth et al., 2010). Interestingly, It has been demonstrated that the intermediate filament proteins, vimentin and desmin, possess lectin-like properties toward O-GlcNAc moieties, supporting physiological interaction between GlcNAc-bearing ligands (and so O-GlcNAc proteins) and lectinic proteins (Ise et al., 2010). The assembly and regular arrangement of the sarcomere results from highly regulated interactions between myofibrillar proteins and structural proteins which are at the origin of a sarcomeric cytoskeleton. It's becoming evident from recent data that a role of O-GlcNAcylation in the organization of the sarcomere should be considered. Since phosphorylation has been demonstrated to be involved in protein-protein interactions, a balance between phosphorylation and O-GlcNAcylation might modulate the dynamics of the sarcomere structural organization. Future investigations will be conducted with aim to determine the role of O-GlcNAcylation in the organization and dynamic of the sarcomere. This work may provide new insights in the understanding of molecular mechanisms of diseases characterized by a disintegration of myofibrils and marked disorganization of the Z band region such as myofibrillar and congenital myopathies.

### Conflict of interest statement

The authors declare that the research was conducted in the absence of any commercial or financial relationships that could be construed as a potential conflict of interest.
